# Synthesis of a Nano-Silver Metal Ink for Use in Thick Conductive Film Fabrication Applied on a Semiconductor Package

**DOI:** 10.1371/journal.pone.0097484

**Published:** 2014-05-15

**Authors:** Lai Chin Yung, Cheong Choke Fei, JS Mandeep, Huda Binti Abdullah, Lai Khin Wee

**Affiliations:** 1 Infineon Technologies (Malaysia) Sdn Bhd (56645-D), Melaka Operations, Free Trade Zone, Batu Berendam, Melaka, Malaysia; 2 Department of Electrical, Electronic & Systems Engineering, Faculty of Engineering & Built Environment, Universiti Kebangsaan Malaysia, Bangi, Selangor, Malaysia; 3 Department of Biomedical Engineering, Faculty of Engineering, University of Malaya, Kuala Lumpur, Malaysia; Dowling College, United States of America

## Abstract

The success of printing technology in the electronics industry primarily depends on the availability of metal printing ink. Various types of commercially available metal ink are widely used in different industries such as the solar cell, radio frequency identification (RFID) and light emitting diode (LED) industries, with limited usage in semiconductor packaging. The use of printed ink in semiconductor IC packaging is limited by several factors such as poor electrical performance and mechanical strength. Poor adhesion of the printed metal track to the epoxy molding compound is another critical factor that has caused a decline in interest in the application of printing technology to the semiconductor industry. In this study, two different groups of adhesion promoters, based on metal and polymer groups, were used to promote adhesion between the printed ink and the epoxy molding substrate. The experimental data show that silver ink with a metal oxide adhesion promoter adheres better than silver ink with a polymer adhesion promoter. This result can be explained by the hydroxyl bonding between the metal oxide promoter and the silane grouping agent on the epoxy substrate, which contributes a greater adhesion strength compared to the polymer adhesion promoter. Hypotheses of the physical and chemical functions of both adhesion promoters are described in detail.

## Introduction

Several conventional printing methods have been developed for forming a conductive track on various printing substrates. Conventional methods such as chemical etching, screen printing and vacuum deposition have been applied to various substrates such as epoxy, polymer and silicon wafer substrates. The chemical etching method is performed by first creating a layer of conductive film on the desired substrate surface, followed by a photolithography step to fabricate the required circuit pattern [1]. This method has been widely used in silicon device fabrication, which requires high-end accurate geometry in the sub-nanometer range. However, chemical etching has a high fabrication cost, including the setup of clean room facilities, which are required for the photolithography process.

The screen printing method is another alternative for patterning and is often used in the newspaper printing industry. Recently, much effort has been made to modify the screen printing method for use in the electronics industry [2]. Radio frequency identification (RFID) is an example of a successful product that requires screen printing technology. In the screen printing process, conductive inks are printed directly on the desired substrate, using a mask to form the conductive tracks.

All of the above-mentioned patterning methods have been widely used in the electronics industry. However, these methods have shortcomings such as complicated processing steps, long process times, low yield rates, the waste of raw materials and, most critically, environmental contamination. Some processes produce waste materials, such as chemical etch solution, chemical cleaning solution or photoresist residue that may cause serious environmental pollution if not properly discarded [3].

To avoid the problems and costs of conventional patterning technologies, many studies have aimed to develop an advanced inkjet printing method for forming a circuit pattern. Inkjet printing technology involves printing the required circuit pattern onto the substrate surface from the printer nozzle using conductive metal ink, with a computer controlling the printing [4].

Inkjet printing technology can not only solve the problems mentioned above but can also further simplify the circuit-design process and shorten the production time. Despite these advantages, inkjet printing technology has not been widely used in the semiconductor industry, mainly due to some unsatisfactory properties of the final printed metal layer [5]. The metal ink applied in the printing process impacts the electrical and mechanical properties of the final circuit. These electrical and mechanical properties have not reached the standards achieved by metal circuits created using conventional methods. To reach these standards, the development of a suitable metal ink is critical.

To print metal ink on the epoxy molding compound that is normally used in the IC encapsulation process, several critical factors must be considered. First, the nano-metal particles should have a diameter of less than 5 nm; these smaller particles have a lower melting temperature than the bulk metal [6], [7], and thus, the metal ink can be widely used on temperature-sensitive substrates that require a lower post-curing-process temperature. Second, to ensure the fluidity of the metal ink, an organic solvent is normally used as an aqueous carrier medium. This medium is selected based on the need for a low post-curing temperature and printability by the inkjet printer. Use of a solvent with a low evaporation temperature ensures that the printed metal track can be fully cured before the epoxy degradation temperature is reached, which normally occurs at approximately 200°C [8].

To ensure the stability of the printing ink during long-term storage, a small percentage of surfactant can be added to metal inks. Normally, the addition of 0.5–2% of a surfactant agent is sufficient to prevent the metal nano-particles from agglomerating at the printer head nozzle during the printing process and to maintain the ink's viscosity [9]. The most commonly used surfactant is Surfynol 465, which can be found in conductive silver ink products [10].

In addition to these considerations, an adhesion promoter is also required to ensure the suitability of the metal ink. The percentage of adhesion promoter added determines the adhesion of the printed metal track on the printing substrate. There is no general adhesion promoter that suits all printing substrates in the electronics market. To create a successful adhesive ink, the specific adhesion promoter must match the printing substrate of interest. In this study, a novel nano-metal ink was fabricated to meet the requirements of the semiconductor industry, specifically for printing on epoxy molding compounds.

## Materials and Methods

Silver nano-powder with diameters ranging from 5–20 nm was purchased from the chemical supplier Sigma-Aldrich, Singapore. The particle sizes were verified using field emission scanning electron microscopy (FESEM). According to the chemical supplier's specifications, the silver powder contained 99.95% silver particles with an additional minor portion of *polyvinylpyrrolidone* (PVP) additives. These PVP additives protect the silver particles from agglomerating during the particle fabrication process.

The alcohol used in the fabrication process was purchased from the same chemical supplier, Sigma-Aldrich. Various types of alcohol are suitable as an aqueous carrier medium, for example, methyl alcohol, ethyl alcohol, n-propyl alcohol, etc. The selection of a suitable candidate is based on its evaporation point and the printing substrate that will be used. In this analysis, methyl alcohol, which has the simplest and shortest chemical C-C bond, was selected due to its lower evaporation temperature. In addition, to improve the adhesion of the printing ink on the epoxy molding substrate, additional additives were needed to form mechanical and chemical linkages between the bonding interfaces. According to the nano-metal ink supplier, the adhesion of silver ink can be improved by using *polyethyleneimine* (PEI) as an adhesion promoter; this promoter also maintains the ink's stability [11]. In addition to improving the interface adhesion, the ability to maintain a high electrical performance must be considered when selecting an adhesion promoter. Using a polymer adhesion promoter at a high concentration may degrade the conductivity performance of the metal ink [12].

In this experiment, a silver ink sample was prepared by mixing the silver nano-powder in methyl alcohol at a weight ratio of 3∶7 with 1–5% of additional adhesion promoter. Two different types of adhesion promoters were evaluated to determine the adhesion strength and electrical properties: PEI as selected as a polymer adhesion promoter, and titanium oxide was selected as a metal oxide adhesion promoter.

During the synthesis of the silver metal ink, mixing the nano-silver powder in the solvent may cause the powder to aggregate. To prevent this phenomenon, a small amount of surfactant was used to improve the ink's wettability. A previous study showed that using 0.5% of the surfactant Surfynol 465 from Air Products is sufficient to maintain the ink's stability and to prevent aggregation of the silver particles.

The DOE shown in table 1 was determined by pre-defining suitable amounts of nano-silver powder and solvent for a fixed amount of 0.5% surfactant (*Surfynol 465*) in the silver ink fabrication. This step is critical to ensure that the silver ink has a suitable viscosity and surface tension to use in an inkjet printer without blocking the printer nozzle. After the ratio of the silver ink contents was defined, different types of adhesion promoters at various weight percentages were added to the initial prepared silver ink solution to assess the interfacial adhesion performance. In this study, 1, 3 and 5 weight % of each adhesion promoter were used in the different sample groups. The use of too much polymer adhesion promoter may degrade the electrical performance of the metal track. To assess the efficiency of the adhesion promoters, a control sample was also prepared without any adhesion promoter.

**Table 1:** The DOE of the silver powder content and the solvent content.

Silver powder content weight %	Solvent weight %	Surfactant weight %
10	90	0.5
20	80	0.5
30	70	0.5
40	60	0.5
50	50	0.5

To assess the performance of the samples, an abrasion shear test and an electrical test were conducted.

## Results and Discussion

The aqueous medium should be optimized to produce suitable ink for inkjet printing. The combination of 30% weight of silver powder and 70% weight of solvent, as shown in the DOE matrix in table 1, was found by a trial run with a Dimatix inkjet printer to be the optimum combination for printing. Thus, this optimum silver ink solution was used to assess the polymer and metal adhesion promoters as shown in table 2 below.

**Table 2:** Sample groups containing different weight percentages and types of adhesion promoter.

Sample identification	Silver content (nano powder)%	Solvent %	Surfactant %	Adhesion promoter %
**A**	silver: 30	methyl alcohol: 70	Surfynol 465: 0.5	PEI: 1.0
**B**	silver: 30	methyl alcohol: 70	Surfynol 465: 0.5	PEI: 3.0
**C**	silver: 30	methyl alcohol: 70	Surfynol 465: 0.5	PEI: 5.0
**D**	silver: 30	methyl alcohol: 70	Surfynol 465: 0.5	TiO_2_: 1.0
**E**	silver: 30	methyl alcohol: 70	Surfynol 465: 0.5	TiO_2_: 3.0
**F**	silver: 30	methyl alcohol: 70	Surfynol 465: 0.5	TiO_2_: 5.0

The silver metal powder was preliminarily mixed with PVP, which functions as a protective agent to prevent agglomeration and as a protective shell to prevent oxidation. A mixture of silver in the aqueous medium with 30% of the nano powder was tested to determine its inkjet-printing efficiency. This concentration exhibited a suitable ink viscosity and surface tension for the inkjet printer. The use of too much silver in the aqueous medium may decrease the performance of the inkjet printer and may cause a blockage in the printing nozzle [12], [13]. In this research, a novel aqueous ink composition for use in inkjet printing was developed, which can hopefully be broadly applied in electronic applications such as in the formation of interconnects on IC packaging and die pads.

The aim of this research was to produce a compatible ink for printing on epoxy molding compound substrates. Preliminary experiments were conducted by printing with ink consisting of an aqueous solution containing a constant amount of Surfynol 465 and different types of adhesion promoters. Two main types of adhesion promoter were studied in this research: PEI (*polyethyleneimine*) and titanium oxide metal. The content percentages of the adhesion promoters used in this experiment were fixed in the range of 1.0 to 5.0%. The incorporation of a larger amount of adhesion promoter might affect the electrical performance of the printed metal track, especially for the polymer adhesion promoter. The epoxy molding substrate was pre-cleaned using an acetone rinse, followed by irradiation with UV/O_3_ to remove all possible contamination sources and to increase the wettability of the epoxy compound [14].

**Figure 1:**
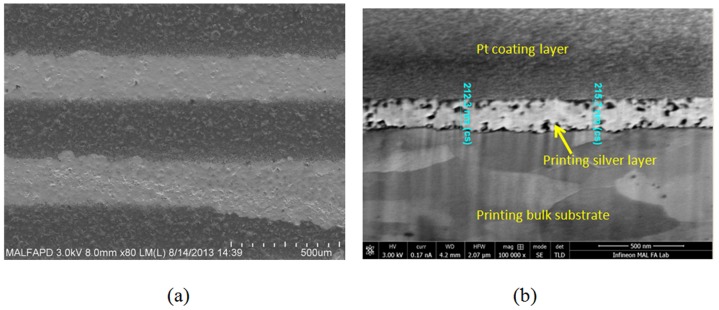
(a) A printed line pattern on an epoxy molding compound that was printed using silver ink. (b) An FIB cross-sectional view of the thickness of the printed silver layer.

The 1^st^ printed metal layer had a thickness of 0.1–0.2 µm, which was achieved with one cycle of printing as shown in figure 1 above. To produce a thicker metal film, multiple overlapping printing steps may be needed. To form a conductive path, the silver particles must be sintered to create a continuous network cluster to enable movement of the electrons. The selection of a sintering method is critical because the sintering process needs to weld all of the particles together while simultaneously preventing degradation of the epoxy substrate at temperatures higher than 200°C. The nano-silver powder in the sub-nanometer range has a low melting temperature compared with the bulk material. To test the sample inks reaction at high temperatures, a hotplate was used to cure the printed silver track pattern, and changes in the phase morphology were observed as the temperature slowly increased from 50°C to 250°C. The silver pattern started to dry out completely when the hotplate temperature reached 180°C. Increasing the hotplate temperature to 250°C caused a color change of the printed silver track from shiny silver to brown.

**Figure 2:**
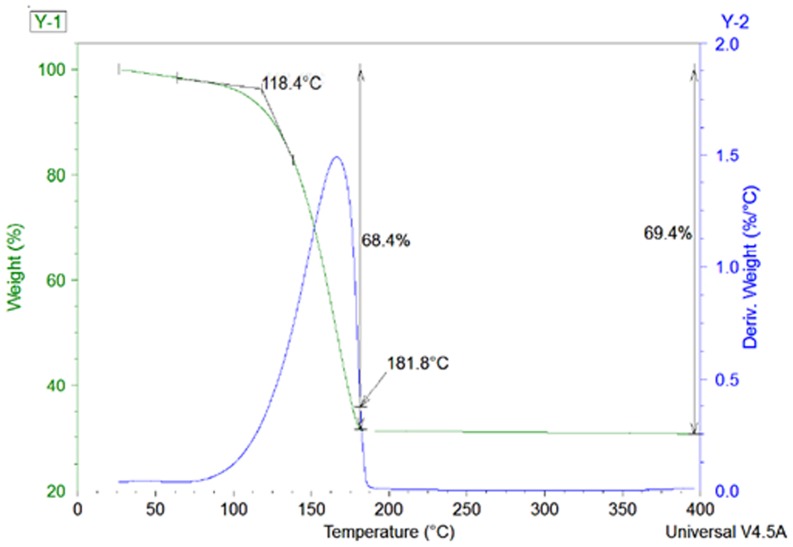
Mass loss versus curing temperature. After the critical curing temperature was determined, all printed samples were cured using a hotplate at 180°C for 1 hour.

Defining the critical curing temperature is important to ensure that the printed pattern is fully cured at the correct temperature. Thermogravimetric analysis (TGA) was conducted to determine the critical curing temperature of the sample ink as shown in figure 2 below. In the TGA, the silver inks showed a mass decrease of approximately 68.4% as the temperature approached 181.8°C. This result suggests that the majority of the solvent had evaporated from the ink. The masses of the remaining silver after sintering match the silver weight contents used in the DOE. The sharp weight-loss gradient curves observed in the TGA graph can be correlated to the actual critical curing temperatures of the inks. This information is critical to ensure that the correct curing temperature is used on the printed layer to form a dense conductive metal cluster.

After the critical curing temperature was determined, all printed samples were cured using a hotplate at 180°C for 1 hour.

The heating process was primarily employed to evaporate the solvent and to force the nano-particles to move closer together to allow for the initial neck formation. To achieve high conductivity, the residues of the other additives, such as the surfactant and the chemical adhesion promoter, must be eliminated, which requires a longer heating time. According to Ashby et al., the sintering process can be described by a grain boundary diffusion model [15]. At 180°C, it is believed that grain boundary diffusion is dominant compared with lattice diffusion [16].

**Figure 3:**
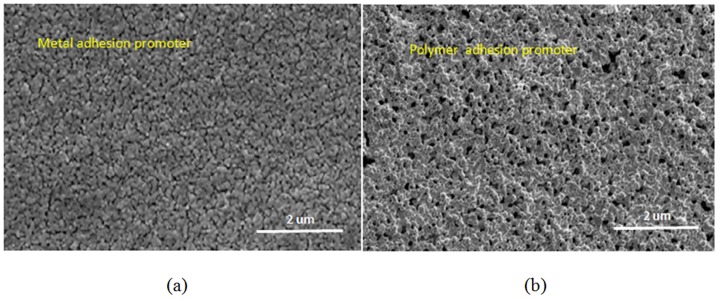
FESEM images of two cured samples with different types of adhesion promoters (5.0%) after sintering on a hotplate at 180°C for 1 hour.

The two different types of adhesive samples were cured using a hotplate for one hour at 180°C. An inspection of the initial surface morphology was conducted using FESEM to assess the structural differences. As shown in figure 3 FESEM images, there were differences in the structural morphology between the printed surfaces built using the polymer and metal adhesion promoters. These differences can be attributed to the chemical reactions occurring between the silver particles and the adhesion promoters. Diluted titanium oxide, the metal adhesion promoter, has a chemical structure state TiO^—^ that can easily be manipulated to form an alloy with the silver particles. Previous studies have shown that this alloy contributes to high-density layer formation after solvent evaporation [17], [18]. In contrast, the polymer adhesion group showed a more porous structure. The PEI acted as a protective shell by binding to the surface of the nano-particles and preventing the silver particles from forming a network cluster.

**Figure 4:**
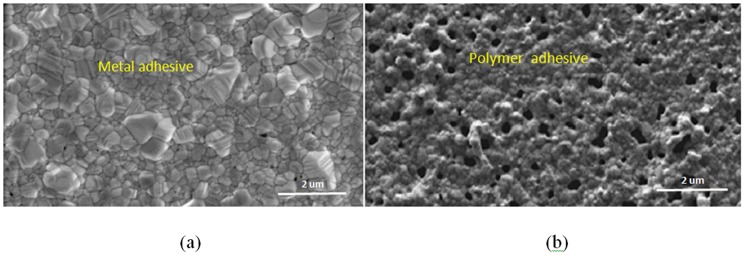
FESEM images of two cured samples with different types of adhesion promoter (5.0%) after further annealing in an oven chamber at 300°C for 96 hours.

After the hotplate treatment, the samples were further annealed using an oven chamber (*Nabertherm*) at 300°C with a forming gas. A forming gas is needed in the baking process to prevent oxidation of the printed silver surface and degradation of the conductivity. After 96 hours of baking, the sample with the metal adhesion promoter showed a denser grain structure compared with the polymer adhesion promoter as shown in figure 4 below. High porosity was observed in the sample with the polymer adhesion promoter, which might be due to remnants of PEI residue between the silver nano-particles that prevent grain boundary diffusion. A greater sample printing thickness would also prevent the PEI from fully evaporating out of the bulk ink.

Further annealing at 300°C will degrade the structure of the epoxy mold compound resulting in the brittleness of the epoxy mold compound substrate. However, the main intention for the annealing process is to ensure that the printed ink is fully cured even though this step is known to degrade the substrate. Therefore, knowing the actual temperature that enables the formation of a dense or cured ink is critical for future development in this process. This will allow simplifying the process of selecting the suitable curing method for the printing inks without degrading the printing substrate applied. [19]

Some PEI remains in the bulk ink because the surface of the printing ink normally cures faster than the interior of the printing ink. This behavior causes the formation of a dense cover layer on top of the printing surface, which is described as the “skinning effect” [20].

The skinning effect caused a blocking effect that prevented the PEI residue from fully evaporating from the interior, and the trapped polymer residue further contributed to the porosity or voids.

**Figure 5:**
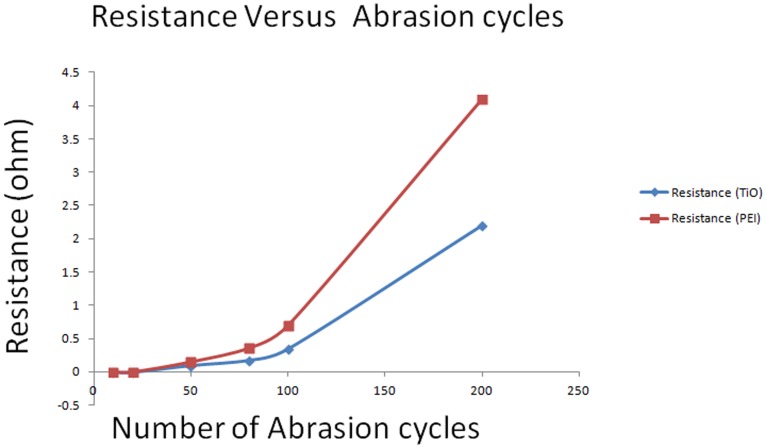
Resistance versus the number of abrasion cycles.

To assess the effectiveness of the adhesion promoter, an abrasion test was conducted using a pin on a disk tribometer [21]. A sample without an adhesion promoter was used as a reference. A constant tip-weight loading was used to assess all of the sample groups. The initial 10-cycle abrasion with a 125-g tip loading showed a minor scratch line on the surfaces of both samples, as shown by FESEM. The abrasion test continued with more test cycles, as shown in Figure 5. To quantitatively monitor the effectiveness of the adhesion promoters, a curve trace was used to assess the changes in the resistance (R) with respect to the number of abrasion test cycles.

Initially, both inks showed approximately the same resistances values, which might be due to the same thickness effect that occurred before the abrasion test. This result may also indicate that both types of adhesion promoter have approximately the same electrical properties. After the abrasion cycles, the ink with the PEI adhesion promoter showed a slightly higher resistance compared with the metal adhesion promoter. This behavior is due to the faster removal rate of the PEI sample compared with the metal ink sample, which resulted in a greater reduction in the bulk volume and larger differences in the resistance. According to Ohm's law, the printing-line length and cross-sectional area or thickness are related to the resistance such that reducing the line thickness proportionally increases the resistance [22].
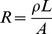



R =  resistance; 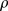  =  resistivity; L =  length; A =  area.

**Figure 6:**
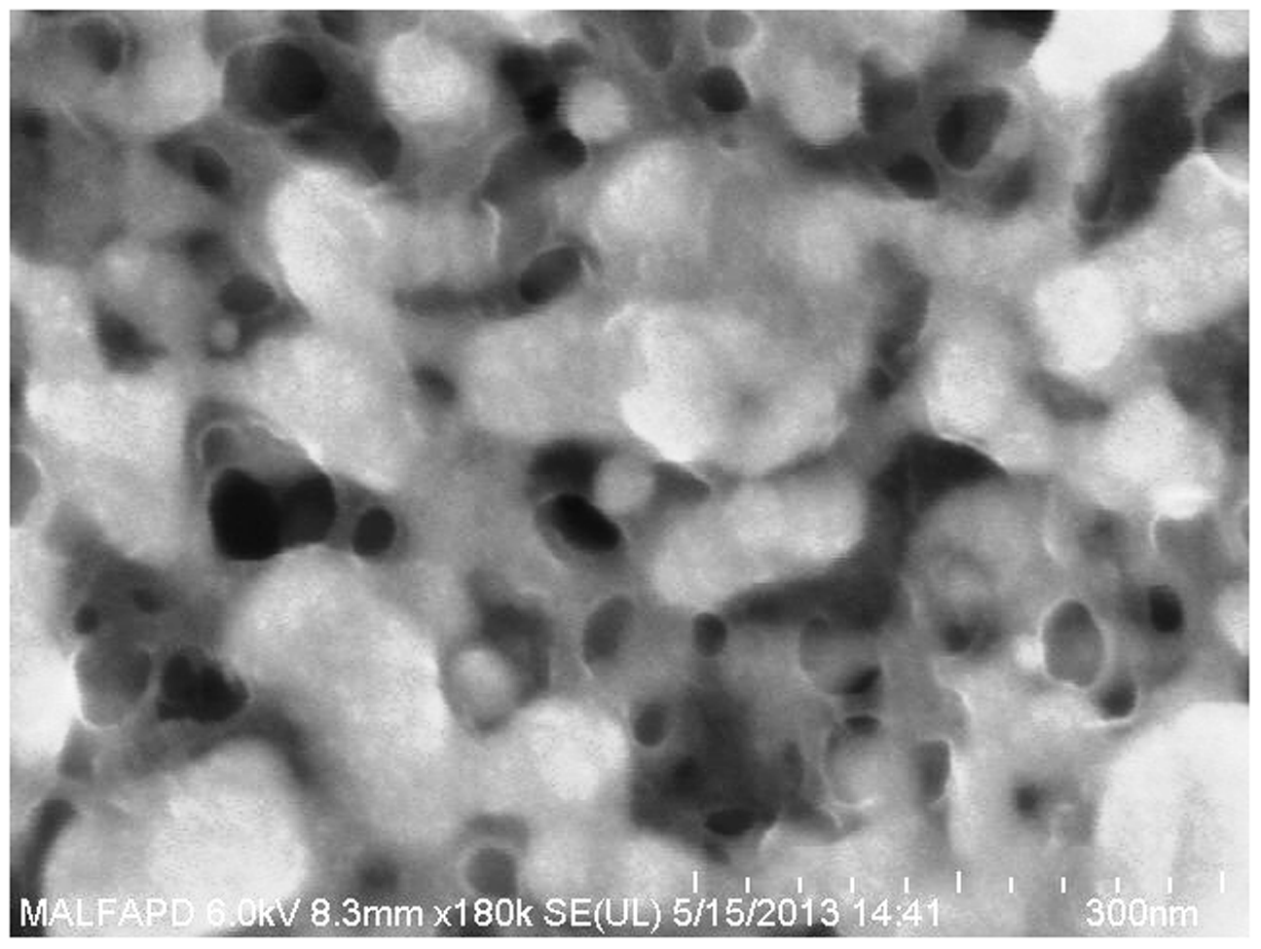
Inter-network of silver particles created from the PEI ink after the heat sintering process.

**Figure 7:**
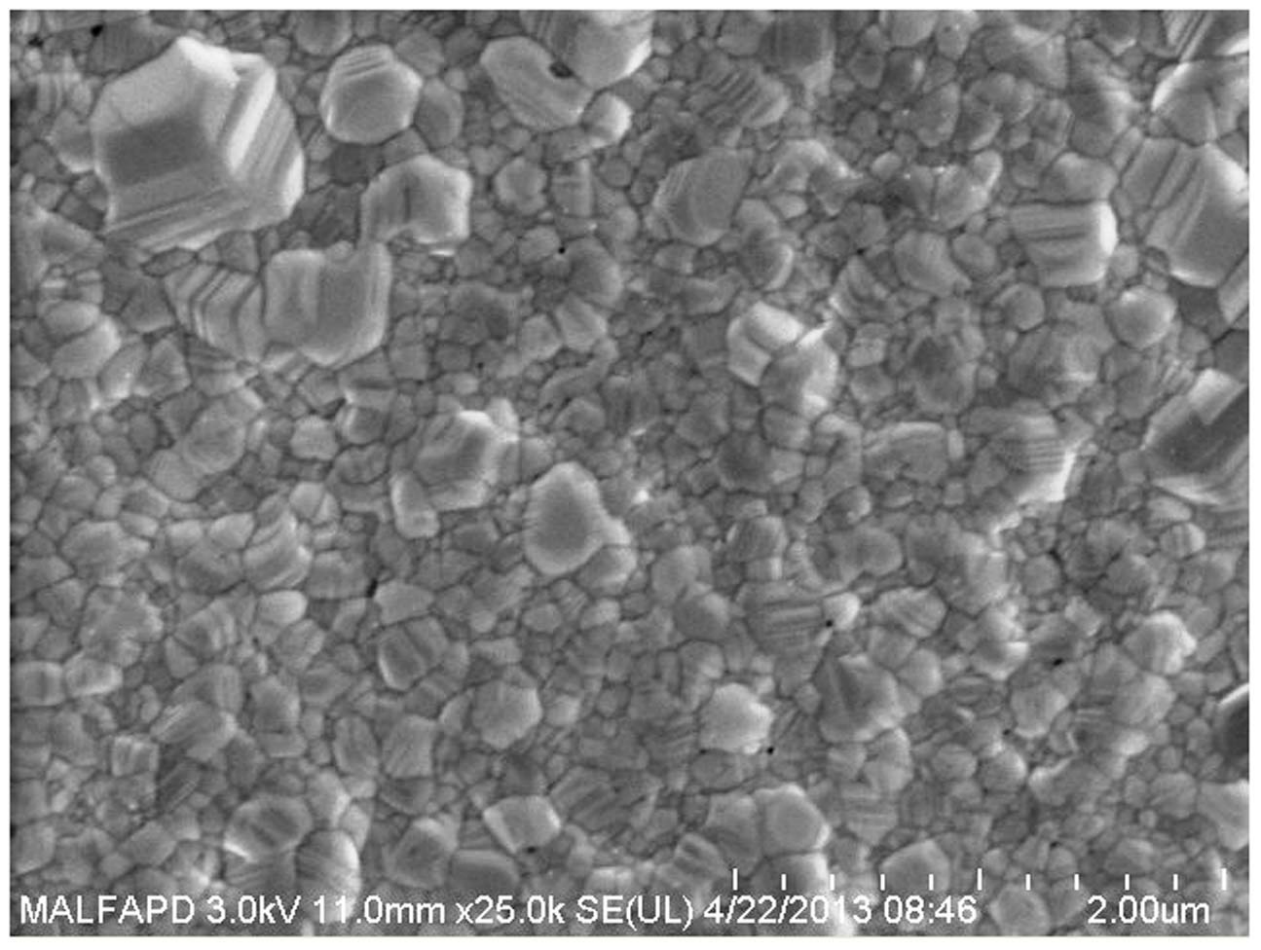
TiAg alloy formation from the TiO_2_ metal ink after the heat sintering process.

The abrasion test showed that the TiO_2_ metal ink had a much greater adhesion strength than the PEI polymer ink. The TiO_2_ adhesion promoter was capable of forming an alloy phase with the silver nano-particles during the sintering process [18], [23], [24], which further increased the bulk metal strength and improved the robustness in the abrasion test. Different behavior was observed for the PEI ink as shown in figure 6, which only showed the formation of silver-atom networking clusters after the sintering process. These inter-networking formations of pure silver particles had much lower bonding strengths compared with the TiAg alloy that could easily be broken by mechanical stress. Figure 6 and figure 7 below showing the cured printed silver structure differences after further heat sintering with oven chamber at 300°C.

**Figure 8:**
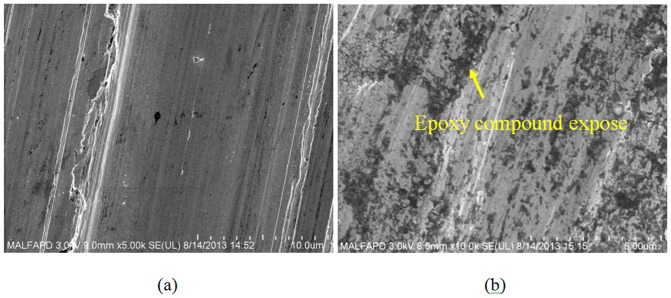
Abrasion test images. (a) TiO_2_ and (b) PEI inks.

To further confirm the bulk printing strength, FESEM was conducted to assess the removal rate of the cured inks after 200 cycles. As shown in Figure 8, the removal rate of the TiO_2_ metal ink was much lower than that of the PEI ink after abrasion tests with the same number of cycles.

In addition to evaluating the bulk bonding strength, the adhesive strength between the printed ink and the epoxy molding substrate was studied. Both the TiO_2_ and PEI inks were further subjected to abrasion tests until the ink on the epoxy molding substrate was completely removed. Performing the abrasion test with the same 125-g weight loading resulted in complete removal of the PEI ink after approximately 800 cycles, whereas the TiO_2_ ink was totally removed after 2500 cycles. These abrasion data confirm that the adhesion of the TiO_2_ ink on the epoxy molding compound is much stronger than that of the PEI ink.

**Figure 9:**
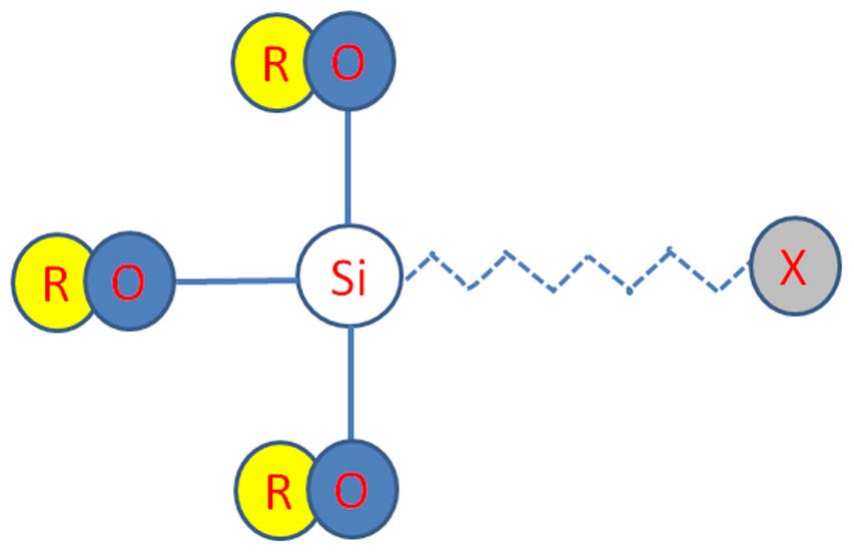
Chemical structure of the silane coupling agent. The R-O structure represents the methoxy or ethoxy functional group. The X- structure represents organic coupling groups such as the epoxy, amino or vinyl group.

This weak adhesion could indicate that the PEI promoter, which contained an amine functional group, only physisorbed to the surface of the epoxy molding substrate by adsorbing tightly to the charged surface without forming any chemical linkages. A different phenomenon was observed with the TiO_2_ ink, which was able to form hydroxyl-group bonds with the epoxy molding substrate. The supplier of the epoxy molding compound indicated that a silane coupling agent was used on the epoxy molding substrate to promote the bonding of organic and inorganic materials. The silane coupling agent as shown in figure 9 below contains reactive functional groups of methoxy or ethoxy that can react with inorganic materials including silica and metal oxides. On the other end of the silane molecule, there are organic-bonding coupling groups such as epoxy, amino and vinyl groups that can react with the synthetic resins in the epoxy molding compound.

**Figure 10:**
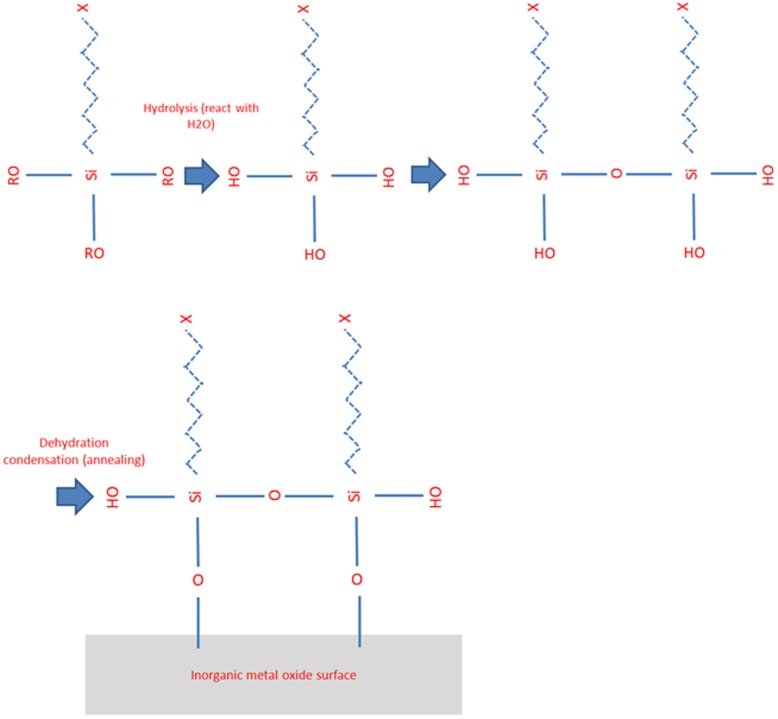
Schematic of the chemical bonding between the TiO^−^ and the silane coupling agent.

With the presence of methoxy or ethoxy functional groups on the epoxy molding substrate, hydroxyl bonds could easily form with the metal adhesion promoter (the TiO_2_ mixture in the metal ink), which could further enhance the interface adhesion strength between the printing ink and the epoxy molding substrate. Figure 10 below showing the details reaction between the TiO_2_ and silane coupling agent.

**Figure 11:**
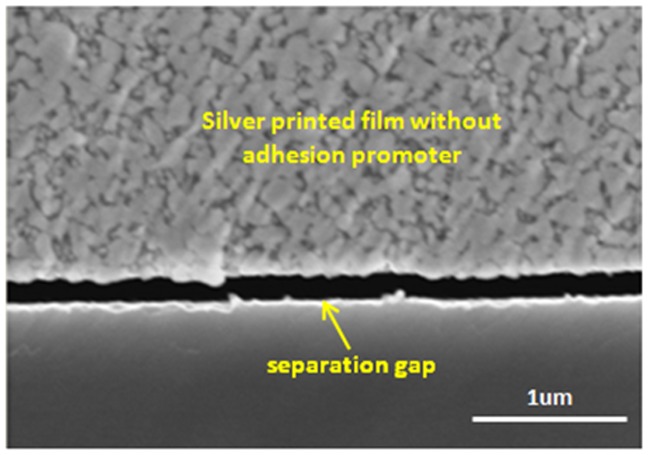
Cross-sectional view of the silver film on the epoxy molding compound when no adhesion promoter was used.

In the sample without any adhesion promoter as shown in figure 11, the adhesion strength of the silver printing ink on the epoxy molding compound after curing with a hotplate at 180°C for 1 hour was very poor. A large separation gap was observed in the cross-sectional view of the sample. The cured silver metal layer could be easily removed from the surface of the epoxy molding compound without the need for mechanical force.

## Conclusion

In this study, a novel conductive ink was developed to meet the needs of semiconductor IC packaging. Different percentages of two adhesion promoters were used. The metal and polymer adhesion promoters that were used did not exhibit any significant differences in mechanical or electrical properties. The novel metal ink meets the requirement of a low curing temperature; it can be cured below 200°C, which is critical for applications to heat-sensitive substrates such as an epoxy molding compound. Abrasion tests showed that the metal adhesion promoter is more suitable than the polymer adhesion promoter for use in IC packaging, which requires a higher adhesion strength between the ink and the epoxy molding substrate. Adhesion of the thick printed metal track to the epoxy molding substrate is critical to ensuring that no internal package delamination occurs during IC package fabrication.
